# Extracellular Histone Released from Leukemic Cells Increases Their Adhesion to Endothelium and Protects them from Spontaneous and Chemotherapy-Induced Leukemic Cell Death

**DOI:** 10.1371/journal.pone.0163982

**Published:** 2016-10-05

**Authors:** Hyun Ju Yoo, Jee-Soo Lee, Ji-Eun Kim, JaYoon Gu, Youngil Koh, Inho Kim, Hyun Kyung Kim

**Affiliations:** 1 Department of Laboratory Medicine, Seoul National University College of Medicine, Seoul, Korea; 2 Cancer Research Institute, Seoul National University College of Medicine, Seoul, Korea; 3 Department of Internal Medicine, Seoul National University College of Medicine, Seoul, Korea; University of Crete, GREECE

## Abstract

**Introduction:**

When leukocytes are stimulated by reactive oxygen species (ROS), they release nuclear contents into the extracellular milieu, called by extracellular traps (ET). The nuclear contents are mainly composed of the histone–DNA complex and neutrophil elastase. This study investigated whether leukemic cells could release ET and the released histone could induce endothelial activation, eventually resulting in leukemic progression.

**Methods:**

The circulating ET were measured in 80 patients with hematologic diseases and 40 healthy controls. ET formation and ROS levels were investigated during leukemic cell proliferation *in vitro*. Histone-induced endothelial adhesion molecules expression and cell survival were measured by flow cytometry.

**Results:**

Acute leukemia patients had high levels of ET, which correlated with peripheral blast count. Leukemic cells produced high ROS levels and released extracellular histone, which was significantly blocked by antioxidants. Histone significantly induced 3 endothelial adhesion molecules expression, and promoted leukemic cell adhesion to endothelial cells, which was inhibited by histone inhibitors (heparin, polysialic acid, and activated protein C), neutralizing antibodies against these adhesion molecules, and a Toll like receptor(TLR)9 antagonist. When leukemic cells were co-cultured with endothelial cells, adherent leukemic cells showed better survival than the non-adherent ones, demonstrating that histone-treated endothelial cells protected leukemic cells from both spontaneous and chemotherapy-induced death.

**Conclusion:**

Our data demonstrate for the first time that extracellular histone can be released from leukemic cells through a ROS-dependent mechanism. The released histone promotes leukemic cell adhesion by inducting the surface expression of endothelial adhesion molecules and eventually protects leukemic cells from cell death.

## Introduction

When leukocytes are stimulated by microorganisms or reactive oxygen species (ROS), they release their nuclear contents into the extracellular milieu, which results in the formation of so-called extracellular traps (ET).[1] The released nuclear contents are mainly composed of the histone–DNA complex and soluble enzymes including neutrophil elastase and cathepsin G. In the innate immune response, the ET are responsible for microorganism entrapment and killing.[2] A recent report has shown that leukemic cell lines release ET upon chemical stimulation.[3] Since leukemic cells are counterparts of normal leukocytes and produce excessive ROS,[4] it is likely that ROS overproduction by these cells induces ET formation.

Histone, the main component of ET, induces inflammation and activates platelets through Toll-like receptor (TLR) activation, thereby exhibiting detrimental effects on host.[5] Recent reports have shown the increased circulating histone levels in inflammatory, autoimmune, and thrombotic disorders.[6, 7] Research into the mechanisms of detrimental effects of ET on the host has primarily focused on inflammatory disorders, while the potential effect of ET in hematologic malignancies remains underappreciated.

The normal endothelium physiologically provides anti-adhesive surface.[8] However, various inflammatory stimuli induce expression of endothelial adhesion molecules such as endothelial cell selectin (E-selectin), intercellular adhesion molecule-1 (ICAM-1), and vascular cell adhesion molecule-1 (VCAM-1), resulting in leukocyte or leukemic cell adhesion to the activated endothelium. Leukemic cell adhesion to endothelium contributes to leukemic progression and chemotherapy resistance.[9–12]

In this study, we hypothesized that circulating histone released from leukemic cells induces endothelial activation, which might protect leukemic cells from spontaneous and chemotherapy-induced death. To test this hypothesis, we measured the circulating levels of 3 ET (histone–DNA complex, cell-free dsDNA, and neutrophil elastase) in patients with several hematologic diseases, and demonstrated a significant correlation between the histone–DNA complex and peripheral blast count. We also demonstrated that several leukemic cell lines released the histone–DNA complex, and this release was inhibited by ROS blockers. Furthermore, histone induced surface expression of endothelial adhesion molecules and thus increased leukemic cell adhesion to endothelium, which was inhibited by neutralizing antibodies against adhesion molecules and by a TLR-9 antagonist. Finally, the adhesion of leukemic cells to histone-activated endothelial cells prevented their spontaneous and chemotherapy-induced death.

## Materials and Methods

### Study populations

A total of 80 patients with hematologic diseases and 40 healthy normal controls were enrolled. The study was approved by the Seoul National University Hospital Institutional Review Board, and written informed consent was obtained from all subjects.

### Cell culture

Human monocytic cell lines (U937, THP-1), a promyelocytic leukemia cell line (HL-60), human endothelial cell line EA.hy926 (hEC), and human umbilical vein endothelial cells (HUVEC) were used. All of them were purchased from ATCC.

For autonomous proliferation, three leukemic cells at a final concentration of 1×10^6^ cells/mL were cultured without media exchange for 5 days. Cell numbers were counted at each time and the culture supernatants were collected. For experiments with inhibitors, Cl-amidine (Calbiochem, San Diego, CA), aurintricarboxylic acid (ATA), quercetin, N-acetyl-l-cysteine (NAC), or 4-amino-2,4-pyrrolidinedicarboxylic acid (APDC; all from Sigma-Aldrich, St. Louis, MO) was added into U937 cell suspensions and the cells were cultured for 5 days.

### Quantitation of ET and ROS activity

The levels of ET were measured by commercial ELISA kits (Cell Death Detection, Roche Diagnostics, Indianapolis, IN; Quant-iT Picogreen dsDNA assay kit, Thermo Fisher Scientific; Human PMN Elastase Platinum, eBioscience, San Diego, CA). Total ROS activity in cell lysates was measured by an OxiSelect *In Vitro* ROS/RNS assay kit (Cell Biolabs, San Diego, CA).

### Confocal microscopy

U937 cells (fresh or cultured for 5 days) were fixed with 4% paraformaldehyde, stained with SYTOX green (Thermo Fisher Scientific) and mounted with Fluoroshield containing DAPI (ImmunoBioScience Corporation, Mukilteo, WA). Images were acquired on an Olympus FluoView FV1000 confocal microscope (Olympus, Tokyo, Japan) with a 100× objective.

### Flow cytometry

hEC or HUVEC were stained with PE-conjugated E-selectin, PE-conjugated ICAM-1, and APC-conjugated VCAM-1 (all from BD Biosciences, Franklin Lakes, NJ).

U937 cells were suspended at a final concentration of 1×10^6^ cells/mL in media and plated on hEC layers pre-treated with 50 μg/mL histone for 1 h. The co-cultured cells were treated with cytosine D–arabinofuranoside (Ara-C; Hospira Pty Ltd., Mulgrave, Australia) for 24 h or without Ara-C for 48 h. Adherent and non-adherent U937 cells were collected separately and stained with FITC-conjugated CD45 (BD Biosciences), PE-conjugated CD105 (BD Biosciences), 7-AAD (Beckman Coulter, Fullerton, CA).

### Adhesion assay

hEC were incubated with or without 50 μg/mL histone for 5 h. U937 cells (1×10^6^ cells/mL) were added onto the hEC layer for 30 min. The non-adherent cells were collected. The adherent round U937 cells were enumerated under a light microscope (Olympus).

For neutralizing histone, histone was pre-mixed with 62.5 μg/mL polysialic acid (Sigma-Aldrich) for 1 h, 100 U/mL heparin (Sigma-Aldrich) for 10 min, and 100 nM activated protein C (APC; Haematologic Technologies Inc., Essex Junction, VA) for 30 min. The mixtures were then added to hEC.

Anti-E-selectin antibody (50 μg/mL), anti-ICAM-1 antibody (10 μg/mL), or anti-VCAM-1 antibody (30 μg/mL) (all from R&D Systems, Minneapolis, MN) was incubated with histone–treated hEC for 10 min. Then U937 cells were added.

Before stimulated with histone, hEC were pre-treated for 1 h with 50 μg/mL isotype-IgG_2a_, anti-TLR2, or anti-TLR4 antibody (all from eBioscience), or 5 μM TLR9 antagonist (ODN TTAGGG; InvivoGen, San Diego, CA).

## Results

### Circulating levels of ET markers in patients with hematologic diseases

The baseline characteristics of the study population are shown in Table 1. Final diagnosis of patients was acute leukemia (n = 21), myeloproliferative neoplasms (MPN, n = 45), and aplastic anemia (n = 14). The acute leukemia group was composed of acute myeloid leukemia (n = 14), acute lymphoblastic leukemia (n = 6), and mixed phenotype acute leukemia (n = 1). MPN patients were subdivided into 2 groups based on absolute neutrophil count (ANC): MPN with neutrophilia (ANC ≥ 7.5×10^9^/L; n = 13) and MPN without neutrophilia (ANC < 7.5×10^9^/L; n = 32). Three ET markers (histone–DNA complex, cell-free dsDNA, and neutrophil elastase) were measured. The level of the histone–DNA complex was significantly higher in the acute leukemia group (311±402) than in the MPN groups either with or without neutrophilia (118±117, *P* = 0.049 and 53±41, *P* = 0.008, respectively). No significant increase in the histone–DNA complex level was observed in patients with aplastic anemia compared with normal control. The circulating levels of cell-free dsDNA and neutrophil elastase were also highest in the acute leukemia group (Table 1). Among patients with MPN, those with neutrophilia exhibited a higher level of neutrophil elastase than those without neutrophilia. Based on the cut-off values (95 percentile of normal control values), positivity for the histone–DNA complex and cell free dsDNA was highest in the acute leukemia group (81.0% and 71.4%, respectively).

**Table 1:** The baseline characteristics and laboratory results of the study populations.

		Normal control (n = 40)	Acute leukemia (n = 21)	MPN with neutrophilia (n = 13)	MPN without neutrophilia (n = 32)	Aplastic anemia (n = 14)
Age (years)		41±10	46±16	60±15*	55±18*	41±22
Male/Female		19/21	15/6	5/8	16/16	7/7
Hemoglobin (g/dL)			8.6±2.1	14.1±4.4	12.9±2.4	8.5±2.3
WBC (x10^9^/L)			43.16±51.85	16.32±3.79	7.48±2.4	2.62±0.98
ANC (x10^9^/L)			5.11±10.44	12.39±3.27	4.82±1.66	0.98±0.53
Platelets (x10^9^/L)			47±37	731±270	706±364	24±15
PT (sec)			13.5±2.0	13.2±1.9	12±1.3	11.1±1.3
aPTT (sec)		33.8±2.3	32±4.4	38.7±5.1	35.9±3.8	31.8±5.0
Antithrombin (%)		96±21	87±17	80±18	86±14	107±19
Fibrinogen (mg/dL)		226±51	309±125	244±68	250±82	280±132
PB blast count (x10^9^/L)		0±0	28.02±40.54	0±0	0±0	0±0
Histone-DNA complex						
	Mean±SD (AU)	30±20	311±402**	118±117*	53±41*	18±18
	Positivity^a^ (n)	2 (5.0%)	17 (81.0%)**	9 (69.2%)**	9 (28.1%)*	0 (0%)
Cell free dsDNA						
	Mean±SD (ng/mL)	62.7±11.7	121.5±62.1**	86.2± 25.5**	75.0± 14.4**	79.1±19.5**
	Positivity^a^ (n)	2 (5.0%)	15 (71.4%)**	7 (53.8%)**	6 (18.8%)	6 (42.9%)**
Neutrophil elastase						
	Mean±SD (ng/mL)	26.1±13.9	126.4±275.9	100.6± 118.0*	45.0± 27.3*	20.2±10.2
	Positivity^a^ (n)	2 (5.0%)	6 (28.6%)*	4 (30.8%)*	4 (12.5%)	0 (0%)

Acute leukemia groups (n = 21) includes acute myeloid leukemia (n = 14), acute lymphoblastic leukemia (n = 6) and mixed phenotype acute leukemia (n = 1)

Values are presented as the mean ± standard deviation or number of subjects. ^a^Positivity was defined when the circulating levels of histone-DNA complex, cell free dsDNA and neutrophil elastase were above the upper normal cutoff of 69 AU, 84.2 ng/mL and 62.7 ng/mL, respectively.

*P <0.05

**P <0.001 vs normal control (*t* test for comparisons of mean values and Chi-square test for comparisons of positivity).

Abbreviations: MPN, myeloproliferative neoplasms; ANC; absolute neutrophil count; PT, prothrombin time; aPTT, activated partial prothrombin time; PB, peripheral blood.

**Fig 1:**
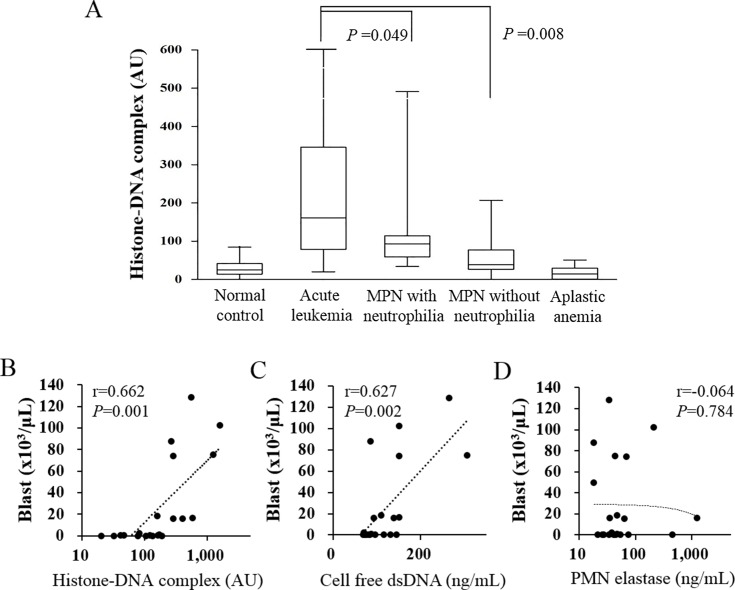
Circulating histone levels are increased in patients with acute leukemia and correlate with peripheral blast counts. (A) Circulating levels of the histone–DNA complex in normal controls (n = 40) and in patients with acute leukemia (n = 21), myeloproliferative neoplasms (MPN) with neutrophilia (n = 13), MPN without neutrophilia (n = 32), and aplastic anemia (n = 14). (B, C, D) Correlation of peripheral blast count with circulating levels of histone–DNA complex, cell-free double-stranded DNA (dsDNA), and neutrophil elastase in acute leukemia patients.

To investigate the factor(s) contributing to the circulating levels of the histone–DNA complex, cell-free dsDNA, and neutrophil elastase, we performed multiple linear regression analysis (Table 2). Peripheral blast count (β = 0.495, SE = 0.001) was the most significant factor contributing to the histone–DNA complex level; the ANC contribution was also significant (β = 0.313, SE = 0.002). Likewise, peripheral blast count (β = 0.731, SE<0.001) and ANC (β = 0.228, SE = 0.001) significantly contributed to the cell-free dsDNA level. ANC (β = 0.860, SE = 0.002) was the only significant contributing factor for neutrophil elastase. In simple correlation analyses, peripheral blast count was significantly correlated with the levels of the histone–DNA complex and cell-free dsDNA, but not with that of neutrophil elastase (Fig 1B–1D). There was a significant correlation between neutrophil elastase and ANC (r = 0.510, *P* = 0.018).

**Table 2:** Multiple regression analyses for determination of contributing factors to histone-DNA complex, cell free dsDNA and neutrophil elastase levels in patients

	Histone-DNA complex	Cell free dsDNA	Neutrophil elastase
Modified R^2^	0.711	0.495	0.677
Age	0.005 (0.836)	-0.001 (0.185)	-0.082 (0.566)
Hemoglobin	-0.120 (5.306)	-0.104 (1.175)	-0.120 (3.595)
ANC	0.313 (0.002)**	0.228 (0.001)*	0.860 (0.002)**
Platelets	0.049 (0.045)	-0.075 (0.010)	-0.153 (0.031)
PT	-0.003 (10.33)	0.116 (2.287)	0.015 (7.000)
Antithrombin	0.122 (0.965)	0.144 (0.214)	0.046 (0.654)
PB blast count	0.495 (0.001)**	0.731 (<0.001)**	-0.091 (0.001)

Values are expressed in regression coefficients β (standard error).

*P <0.05

**P <0.001.

Abbreviations: ANC; absolute neutrophil count; PT, prothrombin time; PB, peripheral blood; dsDNA, double stranded DNA.

### Extracellular histone is released from leukemic cells

**Fig 2:**
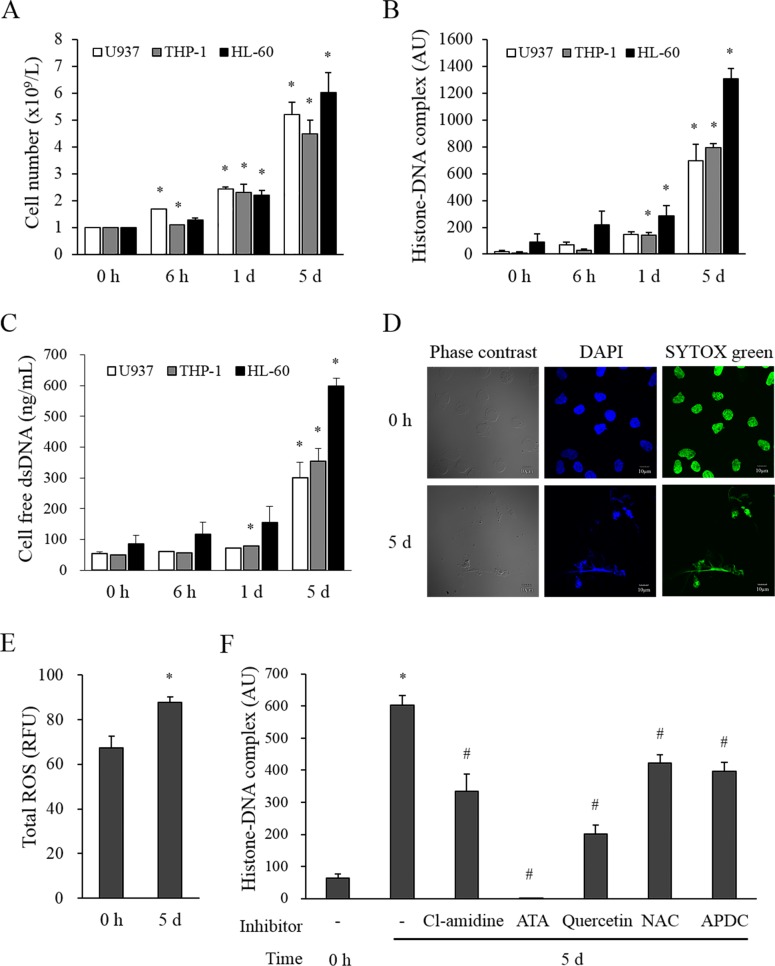
Extracellular traps are released from leukemic cells. Three leukemic cell lines (U937, THP-1, HL-60) were cultured in DMEM with 10% fetal calf serum without medium exchange for 5 days. (A) The numbers of autonomously proliferated cells are expressed as mean ± SEM of 4 experiments. (B, C) The levels of the histone–DNA complex and cell-free dsDNA were measured in the corresponding culture supernatants. (D) U937 cells (fresh or cultured for 5 days) were stained with SYTOX green and DAPI and observed using a confocal laser microscope. Images shown are representative of 3 independent experiments. (E) Total ROS activity was measured in lysates of U937 cells prepared before and after culturing for 5 days (mean±SEM of 3 experiments). (F) U937 cells were pretreated with an inhibitor of extracellular trap formation (200 μM Cl-amidine) or with antioxidants (ATA, 100 μM; quercetin, 100 μM; NAC, 50 μM; or APDC, 100 μM) and the histone–DNA complex levels were measured in the supernatants after 5 day-culture. *P < 0.05 versus each leukemic cell line at 0 h. ^#^*P* < 0.05 versus no inhibitor.

Since peripheral blast count was the most significant contributor to the histone–DNA complex level, it seemed plausible that leukemic blast cells may release the histone–DNA complex into the circulation. To test whether these cells release the histone–DNA complex *in vitro*, we cultured three leukemic cell lines (U937, THP-1, and HL-60) for 5 days without media exchange. The cell number gradually increased (Fig 2A) and the levels of the histone–DNA complex and cell-free dsDNA in the culture supernatant also gradually increased (Fig 2B and 2C). Neutrophil elastase levels in all culture supernatants were less than 0.1 ng/mL (data not shown). To visualize the ET formation, we stained fresh U937 cells and U937 cells cultured for 5 days with SYTOX green and DAPI (Fig 2D). Fresh cells had normal intact round nuclei, whereas those cultured for 5 days showed irregular extracellular dsDNA structures stained with both SYTOX green and DAPI.

We investigated whether ROS production in leukemic cells may induce the release of the histone–DNA complex. As expected, U937 cells cultured for 5 days showed a significantly higher intracellular ROS production than the fresh ones (Fig 2E). Cl-amidine, an inhibitor of peptidylarginine deiminase that induces nuclear chromatin decondensation during ET formation, significantly blocked the histone–DNA complex release (Fig 2F). In addition, four antioxidants (ATA, quercetin, NAC, and APDC) also significantly blocked it to various degrees.

### Histone induces the expression of adhesion molecules on endothelial cell surface

**Fig 3:**
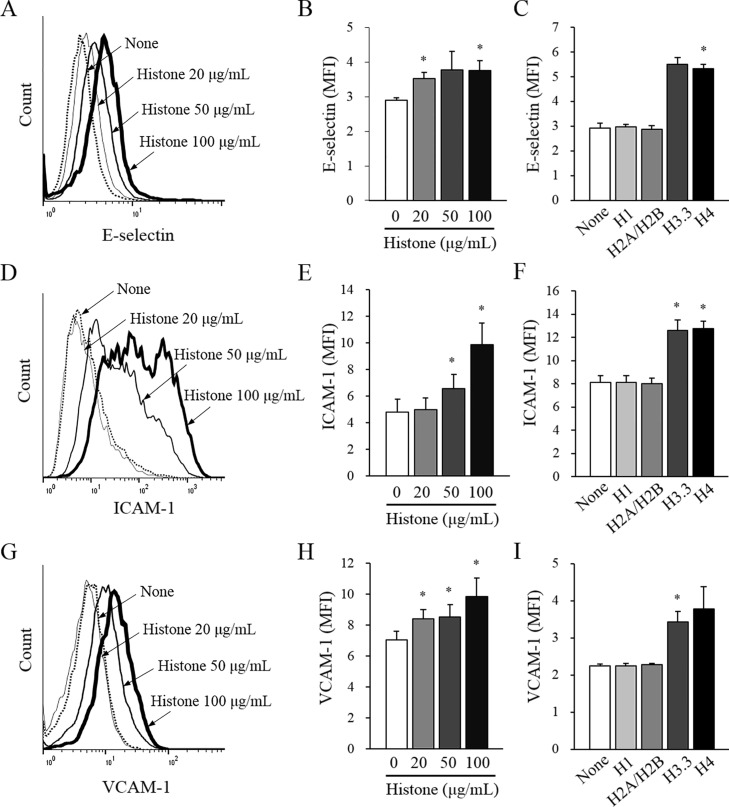
Histone induces surface expression of endothelial adhesion molecules. Endothelial cells (EA.hy926) were cultured with various concentrations of calf thymus histone or 20 μg/mL individual human recombinant histones (H1, H2A/H2B, H3.3, or H4) for 5 h and then the surface expression of (A, B, C) E-selectin, (D, E, F) ICAM-1, and (G, H, I) VCAM-1 were determined by flow cytometry. Histograms are representative of four independent experiments. The bars indicate mean fluorescence intensity (MFI) of each adhesion molecule ± SEM of 4 experiments. *P < 0.05 versus no histone addition.

To explore whether circulating histones activate endothelial cells, the surface expression of endothelial adhesion molecules were measured using flow cytometry. Histone increased the surface expression of all 3 adhesion molecules examined (E-selectin, ICAM-1, and VCAM-1) in a dose-dependent manner (Fig 3A, 3D and 3G, respectively). Among individual histones, H3.3 and H4 significantly increased adhesion molecule expression, whereas H1 and H2A/H2B did not (Fig 3C, 3F and 3I). Histone also increased the expression of these adhesion molecules on the surface of HUVEC (S1 Fig).

### Histone increases leukemic cell adhesion to endothelial cells

**Fig 4:**
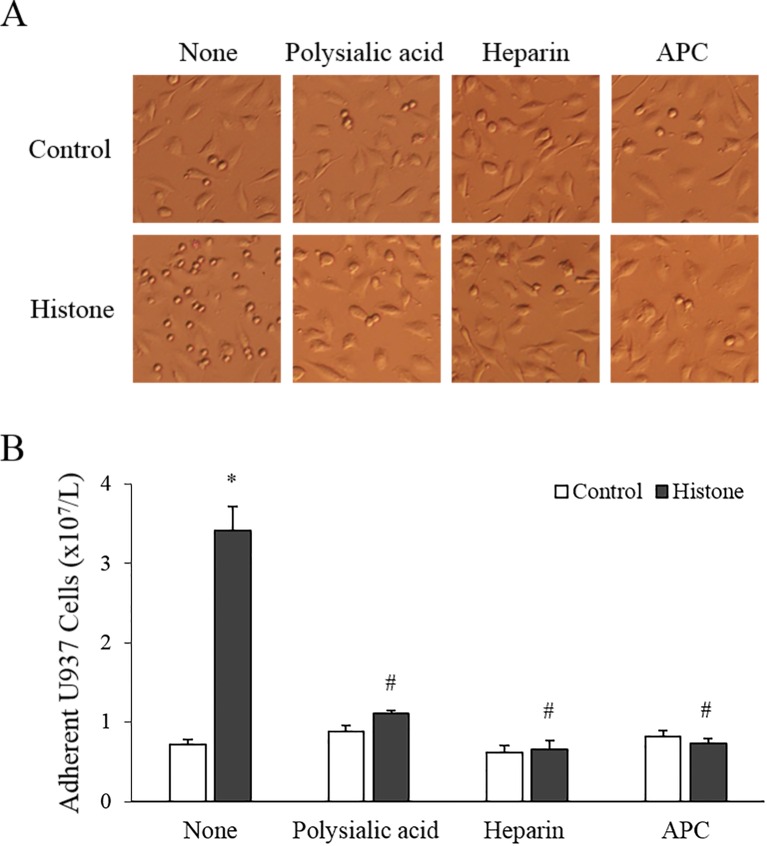
Histone increases leukemic cell adhesion to endothelial cells (EA.hy926; hEC). (A) Calf thymus histone (50 μg/mL) was pre-mixed with 62.5 μg/mL polysialic acid, 100 U/mL heparin, or 100 nM activated protein C (APC) for 1 h, 10 min, and 30 min, respectively, and then added to hEC for 5 h. Then, U937 cells were added onto histone-treated or untreated hEC layers for 30 min. After washing, the adherent small round U937 cells on hEC layers were counted under a microscope (per ×100 field). (B) The numbers of adherent U937 cells are shown as mean ± SEM of 4 experiments. *P < 0.05 versus control; ^#^*P* < 0.05 versus histone-treated.

Since histones enhanced the expression of adhesion molecules on endothelial cell surface, we investigated whether leukemic cells are more likely to adhere to histone-treated endothelial cells than to the untreated ones. As expected, the adhesion of U937 cells was significantly enhanced by histone treatment of endothelial cells (Fig 4A and 4B).

**Fig 5:**
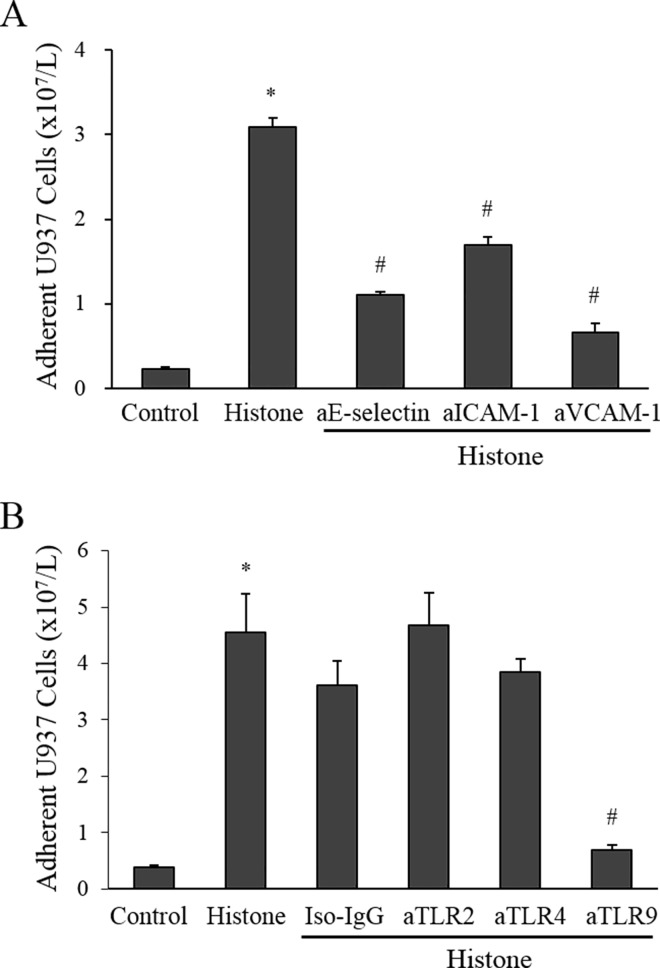
Neutralizing antibodies against adhesion molecules and a Toll-like receptor (TLR)9 antagonist inhibit leukemic cell adhesion to histone-treated endothelial cells (EA.hy926; hEC). (A) Histone-treated hEC were treated with neutralizing antibodies against adhesion molecules (50 μg/mL anti-E-selectin, 10 μg/mL anti-ICAM-1, or 30 μg/mL anti-VCAM-1) for 10 min. Then U937 cells were added and incubated for 30 min, and the adherent U937 cells were counted. (B) Antagonists of TLR2 (50 μg/mL), TLR4 (50 μg/mL) and TLR9 (50 μM) were pre-incubated with hEC before histone stimulation and the adhesion assays were then performed. *P < 0.05 versus control; ^#^*P* < 0.05 versus histone-treated.

Since polysialic acid and heparin bind tightly to histones and APC cleaves histones into fragments,[13–15] we investigated whether these histone inhibitors would reverse the histone effect. Histone pre-incubated with polysialic acid, heparin, or APC failed to increase leukemic cell adhesion when used to treat endothelial cells (Fig 4A and 4B). Accordingly, polysialic acid, heparin, and APC inhibited (albeit partially) adhesion molecule expression on endothelial cells (Figure A in S2 Fig). Neutralizing antibodies against the 3 adhesion molecules (anti-E-selectin, anti-ICAM-1, and anti-VCAM-1) significantly inhibited the leukemic cell adhesion to histone-treated endothelial cells (Fig 5A).

Endothelial cells expressed variable amounts of TLR2, TLR4, and TLR9 on their surface (S3 Fig). To investigate which receptor is involved in the histone-induced increase in endothelial adhesion molecule expression, we pre-incubated endothelial cells with neutralizing antibodies against TLR2, TLR4, or TLR9 before histone stimulation. Anti-TLR2 and anti-TLR4 failed to block the histone-induced increase in endothelial adhesion, whereas anti-TLR9 almost completely blocked it (Fig 5B). Accordingly, the histone-induced expression of adhesion molecules was inhibited by anti-TLR9, but not by anti-TLR2 or anti-TLR4 (Figure B in S2 Fig), indicating that TLR9 is involved in the histone-induced induction of endothelial adhesion molecules.

### Leukemic cells are protected from cell death by their adhesion to histone-treated endothelial cells

**Fig 6:**
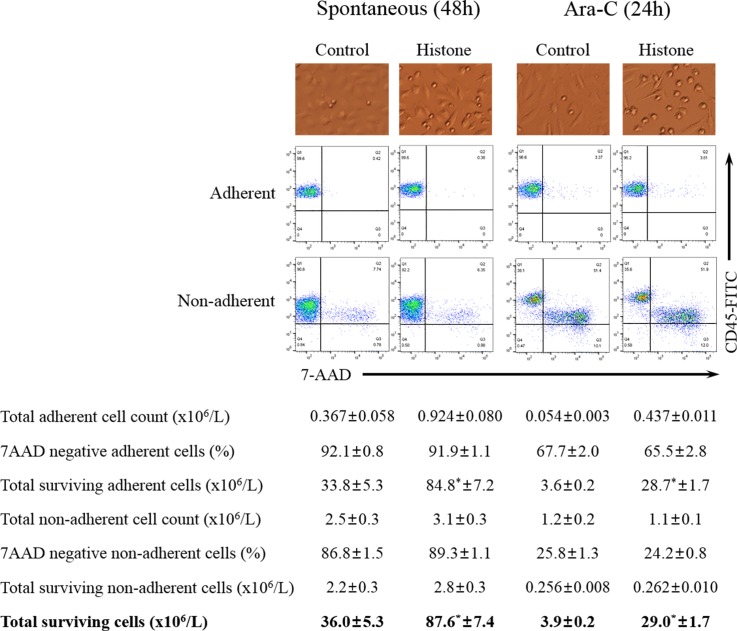
Survival of leukemic cells is increased by their adhesion to histone-treated endothelial cells (EA.hy926). Representative adhesion assay images and flow cytometry plots of CD45 and 7-AAD staining of adherent and non-adherent U937 cells in experiments on spontaneous cell death (48 h) and Ara-C-induced cell death (24 h). Total surviving cells are the sum of total surviving adherent cells and total surviving non-adherent cells. *P < 0.05 versus histone-treated.

To investigate whether spontaneous cell death differed between adherent and non-adherent U937 cells, U937 cells were plated on histone-treated endothelial cells and cultured under serum-deprived conditions for 48 h. Then the adherent and non-adherent U937 cells were collected separately and stained with antibodies against CD45 and CD105, and with 7-AAD. After removal of CD105-positive endothelial cells, both CD45-positive and 7-AAD-negative cells (surviving U937 cells) were counted. When U937 cells were layered onto untreated endothelial cells, 92.1±0.8% of adherent cells survived, which was significantly higher than 86.8±1.5% for non-adherent cells (Fig 6). Likewise, the proportion of surviving U937 cells layered onto histone-treated endothelial cells was significantly higher among the adherent cells (91.9±1.1%) than among non-adherent cells (89.3±1.1%). Since the number of adherent U937 cells was higher when they were layered on histone-treated than on untreated endothelial cells, the absolute number of total surviving U937 cells (adherent + non-adherent) was also higher when histone-treated endothelial cells were used (87.6±7.4 × 10^6^/L) than in the case of untreated endothelial cells (36.0±5.3 × 10^6^/L).

For evaluation of chemotherapy-induced cell death, Ara-C, a commonly used chemotherapeutic agent, was added for 24 h after U937 adhesion to histone-treated or untreated endothelial cells. Similar to the results for spontaneous cell death, the number of surviving U937 cells was significantly higher among adherent cells than among non-adherent cells, and the number of adherent cells was higher when histone-treated endothelial cells were used than in the case of untreated ones (Fig 6). As a result, the total number of surviving U937 cells was significantly higher when they were layered on histone-treated than on untreated endothelial cells.

## Discussion

We demonstrated that the circulating histone level was elevated in patients with acute leukemia, that leukemic cells released extracellular histone *in vitro*, and that this release was blocked by antioxidants. Extracellular histone induced the surface expression of endothelial adhesion molecules and thus promoted the endothelial adhesion of leukemic cells, which protected them from spontaneous or chemotherapy-induced cell death.

Elevated circulating histone levels were reported in various inflammatory, autoimmune, and thrombotic disorders,[6, 7] but not for leukemia. Our study found a high histone level in acute leukemia. Interestingly, the circulating histone level was strongly correlated with the peripheral leukemic blast count. In multiple linear regression analysis, peripheral blast count was the strongest contributor to the histone–DNA complex level. These findings suggest that blast cells are a major source of circulating histone in acute leukemia.

We further investigated whether leukemic cells release the ET *in vitro*. Three leukemic cell lines produced considerable amounts of the histone–DNA complex during culture. Similar to our data, other reports also showed that cultured leukemic cell lines formed ET under certain conditions.[3, 16] However, the detailed mechanism of ET formation is not known. Since it has been reported that ROS are a potent ET inducer and leukemic cells over-produce ROS during proliferation,[4, 16, 17] ROS may potentially cause ET formation by leukemic cells. As expected, our results showed that U937 cells cultured for 5 days produced high levels of intracellular ROS and that the ET formation was significantly inhibited by ROS inhibitors, suggesting that ROS production during leukemic cell proliferation is an important cause of ET formation.

In our study, patients with MPN and neutrophilia had higher levels of the ET markers than patients with MPN without neutrophilia, and ANC was a significant contributor to the ET markers, suggesting that neutrophils are also a source of circulating histone. Among the ET markers, neutrophil elastase, which is secreted from neutrophils, was not correlated with leukemic blast count and was not found in leukemic cell culture supernatants. This finding suggests that neutrophil elastase originated from neutrophils, not from leukemic cells.

Endothelial cells establish a vascular niche that facilitates leukemic cell growth and drug resistance.[18] Several lines of evidence indicate that endothelial activation is essential for leukemia progression.[9–11] Hence, this study focused on the effect of histone on endothelial activation. Histone dose-dependently induced the surface expression of 3 endothelial adhesion molecules, which facilitated leukemic cell adhesion to endothelial cells. The adhesion molecules are usually induced by inflammatory stimuli such as endotoxin.[8] Histone is also known as a strong inflammatory stimulus and its potential receptors have been suggested to be TLR2, TLR4, and TLR9.[5, 19] In our study, TLR2 and TLR4 antagonists did not block the effect of histone, but a TLR9 antagonist did, suggesting that histone induces endothelial activation mainly via TLR9.

To verify whether leukemic cells adhere to endothelial cells through adhesion molecules, we treated histone-induced endothelial cells with neutralizing antibodies against E-selectin, ICAM-1, and VCAM-1. As expected, the 3 antibodies significantly blocked the adhesion, indicating that it is mediated by adhesion molecules. Histone-neutralizing (polysialic acid and heparin) and a histone-degrading (APC) substances almost completely blocked the effect of histone, confirming that the histone used was pure and contained no endotoxin.

When cultured *in vitro*, leukemic cells gradually become apoptotic.[20] Our study demonstrated that leukemic cells adhered to endothelial cells survived better than non-adherent cells; this was true for both spontaneous and chemotherapy-induced cell death, indicating that adhesion is an important survival factor. Our data therefore suggest that the histone-induced adhesion molecules on endothelial cells and increased leukemic cell adhesion protect leukemic cells from spontaneous and chemotherapy-induced death.

Although other stimuli such as direct contact of leukemic cells with soluble angiogenic cytokines have been reported to induce leukemic cell adhesion to endothelial cells,[9–11] our data demonstrate for the first time that another autocrine stimulus, histone, could be released from leukemic cells and promote their adhesion to endothelial cells by inducing endothelial adhesion molecules. These effects would eventually promote leukemia progression through the rescue of leukemic cells from spontaneous and chemotherapy-induced cell death.

## Supporting Information

S1 Fig Histone induces adhesion molecules. (PDF)

S2 Fig Effects of histone inhibitors on histone-induced endothelial adhesion molecule expression. (PDF)

S3 Fig Surface expression of Toll-like receptor (TLR)s. (PDF)
