# Supplementary material for: Extracellular Histone Released from Leukemic Cells Increases Their Adhesion to Endothelium and Protects them from Spontaneous and Chemotherapy-Induced Leukemic Cell Death
Source: PLoS One. 2016 Oct 5;11(10):e0163982. doi: 10.1371/journal.pone.0163982 (PMC5051947; doi:10.1371/journal.pone.0163982)
Supplement: S1 Fig — (PDF) [file pone.0163982.s001.pdf]

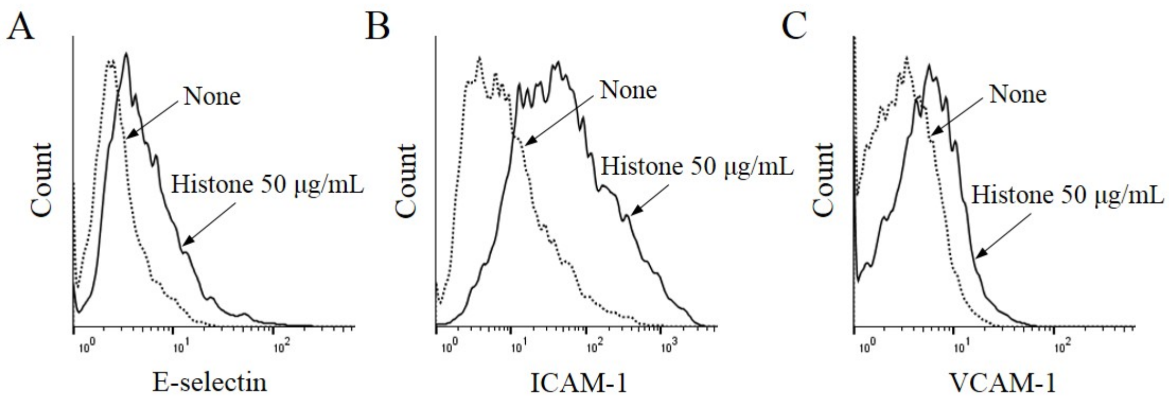

S1 Fig. Histone induces adhesion molecules. Human umbilical vein endothelial cells were treated with 50 μg/mL calf thymus histone for 5 h and then the surface expression of E-selectin, ICAM-1, and VCAM-1 was determined by flow cytometry. Histograms are representative of three independent experiments.
