# Supplementary material for: Extracellular Histone Released from Leukemic Cells Increases Their Adhesion to Endothelium and Protects them from Spontaneous and Chemotherapy-Induced Leukemic Cell Death
Source: PLoS One. 2016 Oct 5;11(10):e0163982. doi: 10.1371/journal.pone.0163982 (PMC5051947; doi:10.1371/journal.pone.0163982)
Supplement: S2 Fig — (PDF) [file pone.0163982.s002.pdf]

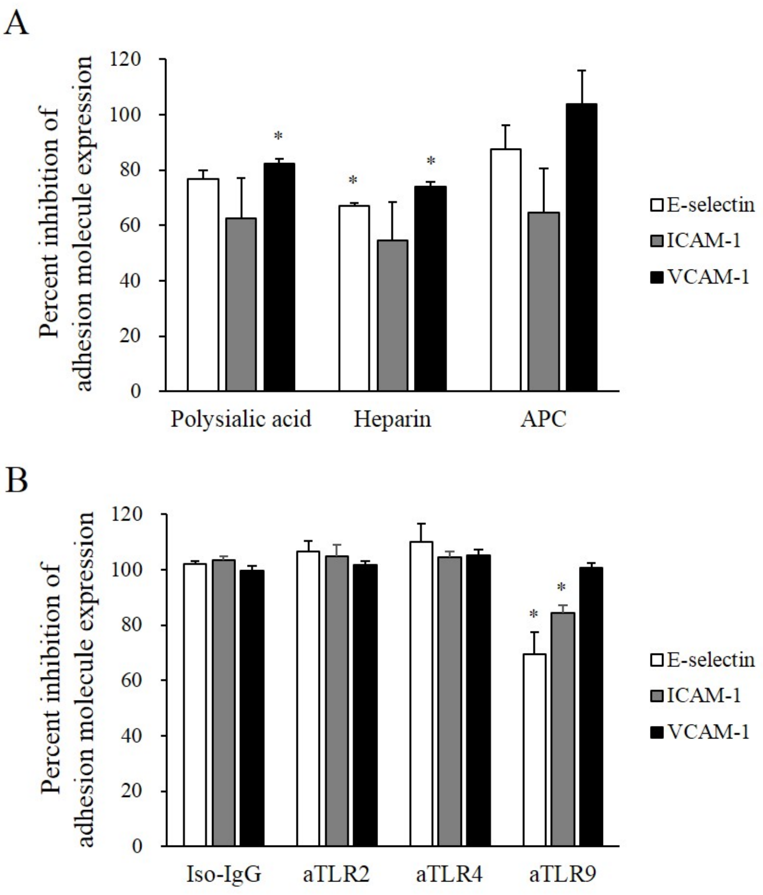

S2 Fig. Effects of histone inhibitors on histone-induced endothelial adhesion molecule expression. (A) Calf thymus histone (50 μg/mL) was pre-mixed with polysialic acid, heparin, or activated protein C (APC) for 1 h, 10 min and 30 min, respectively. The mixtures were then added to endothelial cells (EA.hy926; hEC) for 5 h and the surface expression of E-selectin, ICAM-1, and VCAM-1 was determined by flow cytometry. The percent inhibition of mean fluorescence intensity of each adhesion molecule compared to no inhibitor addition is shown as mean ± SEM of 4 experiments. (B) Antibodies against Toll-like receptor (TLR)2, TLR4, and TLR9 were incubated with hEC before histone stimulation. The above surface staining was then performed. $^{*}P < 0.05$ versus no inhibitor addition (A) or isotype control (B).
