# Supplementary material for: Extracellular Histone Released from Leukemic Cells Increases Their Adhesion to Endothelium and Protects them from Spontaneous and Chemotherapy-Induced Leukemic Cell Death
Source: PLoS One. 2016 Oct 5;11(10):e0163982. doi: 10.1371/journal.pone.0163982 (PMC5051947; doi:10.1371/journal.pone.0163982)
Supplement: S3 Fig — (PDF) [file pone.0163982.s003.pdf]

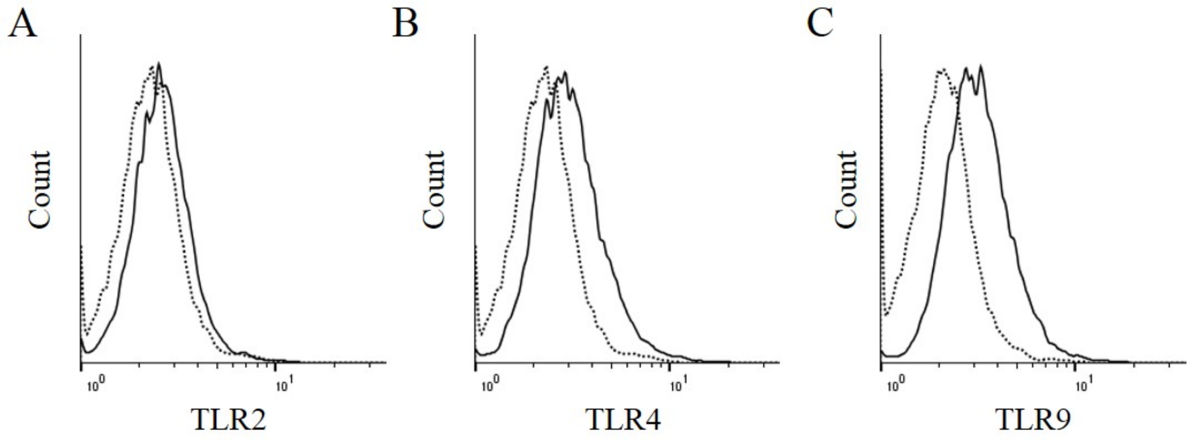

S3 Fig. Surface expression of Toll-like receptor (TLR)s. Surface expression of TLR2, TLR4, and TLR9 on endothelial cells (EA.hy926) measured by flow cytometry. Histograms are representative of three independent experiments.
